# Missing paternal demographics: A novel indicator for identifying high risk population of adverse pregnancy outcomes

**DOI:** 10.1186/1471-2393-4-21

**Published:** 2004-11-13

**Authors:** Hongzhuan Tan, Shi Wu Wen, Mark Walker, Kitaw Demissie

**Affiliations:** 1 OMNI Research Group, Department of Obstetrics & Gynecology, University of Ottawa, Faculty of Medicine, 501 Smyth Rd, Ottawa, K1H 8L6, Canada; 2 Clinical Epidemiology Program, Ottawa Health Research Institute, 501 Smyth Rd, Ottawa, K1H 8L6, Canada; 3 School of Public Health, Central South University, Changsha, Hunan 410008, P. R. China; 4 Division of Epidemiology, School of Public Health, University of Medicine and Dentistry of New Jersey, Piscataway, New Jersey, USA

## Abstract

**Background:**

One of every 6 United Status birth certificates contains no information on fathers. There might be important differences in the pregnancy outcomes between mothers with versus those without partner information. The object of this study was to assess whether and to what extent outcomes in pregnant women who did not have partner information differ from those who had.

**Methods:**

We carried out a population-based retrospective cohort study based on the registry data in the United States for the period of 1995–1997, which was a matched multiple birth file (only twins were included in the current analysis). We divided the study subjects into three groups according to the availability of partner information: available, partly missing, and totally missing. We compared the distribution of maternal characteristics, maternal morbidity, labor and delivery complications, obstetric interventions, preterm birth, fetal growth restriction, low birth weight, congenital anomalies, fetal death, neonatal death, post-neonatal death, and neonatal morbidity among three study groups.

**Results:**

There were 304466 twins included in our study. Mothers whose partner's information was partly missing and (especially) totally missing tended to be younger, of black race, unmarried, with less education, smoking cigarette during pregnancy, and with inadequate prenatal care. The rates of preterm birth, fetal growth restriction, low birth weight, Apgar score <7, fetal mortality, neonatal mortality, and post-neonatal mortality were significantly increased in mothers whose partner's information was partly or (especially) totally missing.

**Conclusions:**

Mothers whose partner's information was partly and (especially) totally missing are at higher risk of adverse pregnant outcomes, and clinicians and public health workers should be alerted to this important social factor.

## Background

Pregnancy outcomes are not only important measures of health status of the mothers and infants, but are important measures of socioeconomic development in a society. For example, infant mortality has been considered the single most comprehensive measure of the health and wealth in a society [1,2]. Both maternal and paternal characteristics, such as age, race, marital status, education, and cigarette smoking [3-5], are important determinants of pregnancy outcomes. However, partner information is often missing in routine vital statistics data. For example, about 17% of United Status birth certificates contain no paternal information and more than 40% of the babies born to adolescent women have no information on the paternal age in the birth certificate [6]. There might be important differences in the pregnancy outcomes between mothers with versus those without paternal information. Detailed description of these differences may benefit the clinicians and public health workers who serve high risk pregnant women. We conducted extensive search of the literature and have not been able to locate a single study on this issue.

Epidemiologic studies based on a data with missing information on exposure, outcomes, and potential confounders can bias the study results, unless the missing occurred randomly [7]. Previous studies on missing data [8-11] have focused on the impact of missing information on study results and the methods to treat the missing variables. The main objective of the current study is to describe the distribution of maternal characteristics and pregnancy outcomes in mothers whose partner's information was partly or totally missing. We also attempted to assess the potential source of missing partner information and its impact on outcomes, by exploring the reasons of differences between partly missing versus totally missing.

We used a large twin registry data in the United States to examine this issue. This data has been used in several peer-reviewed studies [12-14]. The reasons of using data on twins were two folds. First, adverse pregnancy outcomes are more common in twins than in singletons. Therefore, it is easier to detect the difference in adverse pregnancy outcomes in twins than in singletons. Second, because linkage for multiple births requires an examination and processing of the recorded variables, it will increase the validity of the administrative data.

## Methods

We used the matched multiple birth file created by the National Center for Health Statistics (NCHS), Centers for Disease Control and Prevention [15]. The multiple birth file in 1995–1997 in the United States were linked infant deaths information according to uniform coding specifications, which have undergone vigorous editing and reviewing during the process of the record linkage. Sets of multiples in the 1995–1997 birth file were matched by plurality, state and county of occurrence of delivery, mother's date of birth, date of last menstrual period (LMP), number of prenatal visits, level of education, weight gain during pregnancy, and date of delivery. The matching was successful for 98% of the multiple sets [15]. Available study variables in the database include socio-demographic information of the parents, maternal life-style factors such as smoking during pregnancy, obstetric history, complications of the pregnancy, labor and delivery, birth weight, gestational age, and other infant outcome variables. Only complete twin sets were included in the current study, with twin set as the unit of analysis in comparison of maternal characteristics and outcomes, and randomly selected one twin in each twin sets as the unit of analysis for fetal and neonatal outcomes.

There were two variables for the partners in the data: partner's age and race. The eligible study subjects were divided into three groups according to the availability of the two variables of the partners: both available (group 1), one missing (group 2), and both missing (group 3).

We first described the distribution of the maternal characteristics (age, race, marital status, education, cigarette smoking, place of birth, and prenatal care service) of the three study groups. The prenatal care service was defined as adequate, intermediate, and inadequate according to the time of prenatal care visit initiation and the number of prenatal visit using the method described by Kessner et al [16]. If the first prenatal care initiated at third trimester, or the times of prenatal visit less than 5 in 34 gestation weeks, 4 in 32–33 weeks, 3 in 30–31 weeks, or 2 in 22–29 weeks, the prenatal care service was defined as inadequate; if the first prenatal care initiated at first trimester, and the times of prenatal visit more than 8 in 36 gestation weeks, 7 in 34–35 weeks, 6 in 32–33 weeks, 5 in 30–31 weeks, 4 in 26–29 weeks, 3 in 22–25 weeks, or 2 in 18–21 weeks, the prenatal care service was defined as adequate; and the remainders were defined as intermediate. We compared the rates of maternal medical complications (anemia, cardiac disease, acute or chronic lung disease, diabetes, genital herpes, hydramnios, oligohydramnios, hemoglobinopathy, chronic hypertension, pregnancy-associated hypertension, eclampsia, incompetent cervix, renal disease, RH sensitization, and uterine bleeding), labor and delivery complications (febrile (any temperature reading >38°C), meconium, premature rupture of membrane (>12 hours), abruption placenta, plancenta previa, seizures during labor, precipitous labor (2^nd ^stage <3 hours), prolonged labor (2^nd ^stage >20 hours), dysfunctional labor, breech, malpresentation, cephalopelvic disproportion, cord prolapse, anesthetic complications, and fetal distress), and obstetric interventions (cesarean section, vacuum / forceps, and labor inductions) among the three study groups. Finally we compared the rates of fetal and neonatal mortality and morbidity among the three study groups. We used the Chi-square test to test the difference of all rates among the three study groups, used the normal approximate method to calculate the 95% confidence interval of the rates of maternal medical complications, labor and delivery complications, obstetric interventions, and the fetal and neonatal mortality and morbidity. We calculated the relative risk in group 2 and group 3 compared with group 1 for all the outcomes mentioned above. Fetal death was defined as stillbirth weighing 500 grams or more, or if weight was unknown, 20 completed gestation weeks or more. Neonatal death was defined as live-born infant died at 0–27 days of age, and post-neonatal death was defined as those died at 28–364 days of age. Preterm birth (PTB) was defined as gestational age < 37 weeks. Fetal growth restriction (FGR) was defined as less than 10th percentile of birth weight-for-gestational-age z score. The birth weight-for-gestational-age z score was calculated using the following formula:

Z = (observed birth weight - mean of birth weight)/SD

Where mean and SD was based on all infants in the database, stratified by gender and gestational week. Low birth weight (LBW) was defined as birth weight <2500 grams. Low Apgar score was defined as five minute Apgar score < 7. Anemia was defined as hemoglobin <13. Other fetal and infant outcomes examined included birth injury, hyaline membrance disease, meconium aspiration syndrome, assisted ventilation (<30 minutes and > = 30 minutes), seizures, central nervous system anomalies, circulatory / respiratory anomalies, digestive system anomalies, urogenital system anomalies, musculoskeletal / integumental anomalies, and chromosomal anomalies.

## Results

There were 304466 twins (152233 twin pairs) in the database. Of which, father's age and race were recorded in 259302 twins (129651 twin pairs, 85.17%, group 1), father's age or race was missing in 6036 twins (3018 twin pairs, 1.98%, group 2), and both father's age and race were missing in 39128 twins (19564 twin pairs, 12.85%, group 3).

Table 1 describes the distribution of maternal characteristics and prenatal care service among the three study groups. The proportions of teenage, blacks, unmarried, lower education, cigarette smoking during pregnancy, and inadequate perinatal care service were significantly higher in group 2 and (especially) group 3 as compared with group 1 (Table 1).

**Table 1:** The distribution (%) of baseline maternal characteristic among three study groups

		Partner information available (N = 129651)	Partner information partly missing (N = 3018)	Partner information totally missing (N = 19564)
Age*	<20 y	5.1	13.7	21.4
	20 y-	75.9	72.2	69.4
	35 y-	19.0	14.1	9.2
Race*	white	84.0	69.0	46.1
	Black	12.1	26.3	51.3
	Other	3.9	4.7	2.6
Marital status*	Married	82.6	39.0	4.1
	Unmarried	17.4	61.0	95.9
Education*	<12 y	13.1	28.4	36.1
	12 y	30.0	31.2	39.9
	13–15 y	24.1	15.2	15.9
	16 y	19.6	5.8	2.9
	>16 y	13.2	19.4	5.2
Cigarette smoking*	Yes	8.1	14.9	19.1
	No	70.5	55.8	69.5
	Not available#	21.3	29.4	11.4
Prenatal care service*	Adequate	79.4	55.5	54.1
	Mediate	17.8	37.0	34.6
	Inadequate	2.7	7.5	11.3
Place of birth*	Native	84.5	72.7	88.1
	Foreign	15.5	27.3	11.9

*Compared group 2 and group 3 with group 1 with Chi-square test, p < 0.001

# The state of California did not send data on smoking, and all births to residents in California were classified as not available.

Table 2 compares the rate of maternal morbidity, labor and delivery complications, and obstetric interventions among the three study groups. The rates of anemia, acute or chronic lung disease, hemoglobinopathy, chronic hypertension, eclampsia, renal disease, meconium, premature rupture of membrane, abruption placenta, and precipitous labor were significantly higher, and the rates of cardiac disease, diabetes, RH sensitization, placenta previa, dysfunctional labor, cephalopelvic disproportion, cesarean section, vacuum / forceps delivery, and induction were significantly lower in group 2 and (especially) group 3 than in group 1 (Table 2).

**Table 2:** Comparison of maternal medical complication, labor complication, and obstetric intervention among three study groups (%, 95%CI)

	Partner information available (N = 129651)	Partner information partly missing (N = 3018)	Partner information totally missing (N = 19564)
Medical risk factors			
Anemia	2.81 (2.72 – 2.90)	4.27 (3.55 – 5.00)*	4.67 (4.37 – 4.96)*
Cardiac disease	0.64 (0.60 – 0.69)	0.40 (0.17 – 0.62)	0.44 (0.35 – 0.53)*
Acute or chronic lung disease	0.90 (0.85 – 0.95)	1.36 (0.95 – 1.77)*	1.64 (1.46 – 1.82)*
Diabetes	3.46 (3.36 – 3.56)	3.02 (2.40 – 3.63)	2.15 (1.94 – 2.35)*
Genital herpes	0.76 (0.71 – 0.80)	1.06 (0.69 – 1.43)	0.82 (0.70 – 0.95)
Hydramnios / Oligohydramnios	1.87 (1.80 – 1.94)	2.95 (2.35 – 3.55)*	2.08 (1.88 – 2.28)
Hemoglobinopathy	0.07 (0.06 – 0.08)	0.17 (0.02 – 0.31)	0.19 (0.13 – 0.26)*
Chronic hypertension	0.90 (0.85 – 0.95)	0.89 (0.56 – 1.23)	1.20 (1.04 – 1.35)*
Pregnancy-associated hypertension	7.61 (7.46 – 7.75)	6.39 (5.52 – 7.27)*	7.38 (7.01 – 7.75)
Eclampsia	0.94 (0.89 – 0.99)	1.39 (0.97 – 1.81)	1.22 (1.07 – 1.38)*
Incompetent cervix	0.81 (0.76 – 0.85)	0.80 (0.48 – 1.11)	0.72 (0.60 – 0.84)
Renal disease	0.28 (0.25 – 0.31)	0.56 (0.30 – 0.83)	0.40 (0.31 – 0.49)*
RH sensitization	069 (0.64 – 0.73)	0.53 (0.27 – 0.79)	0.49 (0.39 – 0.58)*
Uterine bleeding	1.10 (1.04 – 1.16)	0.83 (0.50 – 1.15)	0.97 (0.83 – 1.10)
Complication of labor			
Febrile	1.36 (1.30 – 1.43)	1.36 (0.95 – 1.77)	1.48 (1.31 – 1.65)
Meconium	1.37 (1.31 – 1.43)	2.09 (1.58 – 2.69)*	1.92 (1.73 – 2.11)*
Premature rupture of membrane (>12 h)	6.34 (6.21 – 6.47)	7.19 (6.27 – 8.11)	7.91 (7.53 – 8.29)*
Abruptio placenta	1.16 (1.10 – 1.22)	1.03 (0.67 – 1.39)	1.44 (1.27 – 1.61)*
Placenta previa	0.47 (0.43 – 0.51)	0.50 (0.25 – 0.75)	0.30 (0.22 – 0.38)*
Seizures during labor	0.05 (0.04 – 0.06)	0.07 (0.03 – 0.16)	0.08 (0.04 – 0.12)
Precipitous labor (<3 hours)	1.42 (1.36 – 1.49)	1.29 (0.89 – 1.70)	1.86 (1.67 – 2.04)*
Prolonged labor (>20 hours)	0.56 (0.52 – 0.60)	0.63 (0.35 – 0.91)	0.52 (0.42 – 0.62)
Dysfunctional labor	2.34 (2.26 – 2.42)	1.52 (1.09 – 1.96)*	1.97 (1.77 – 2.16)*
Breech / malpresentation	21.3 (21.0 – 21.5)	21.4 (20.0 – 22.9)	21.4 (20.8 – 22.0)
Cephalopelvic disproportion	0.90 (0.85 – 0.95)	0.76 (0.45 – 1.07)	0.52 (0.42 – 0.62)*
Cord prolapse	0.55 (0.51 – 0.59)	0.56 (0.30 – 0.88)	0.65 (0.54 – 0.77)
Anesthetic complications	0.07 (0.06 – 0.09)	0.10 (0.01 – 0.21)	0.09 (0.05 – 0.13)
Fetal distress	3.20 (3.10 – 3.29)	3.45 (2.79 – 4.10)	3.44 (3.18 – 3.70)
Obstetric intervention			
Cesarean	52.0 (51.7 – 52.3)	46.1 (44.7 – 48.2)*	47.5 (46.8 – 48.2)*
Vacuum / Forceps	6.76 (6.62 – 6.89)	5.40 (4.59 – 6.21)*	4.46 (4.17 – 4.75)*
Induction	13.0 (12.8 – 13.2)	11.9 (10.7 – 13.0)	10.2 (9.8 – 10.6)*

*P < 0.05 for group 2 or group 3 compared with group 1

Table 3 compares the fetal and neonatal mortality and morbidity among the three study groups. The rates of PTB, FGR, LBW, Apgar score <7, fetal mortality, neonatal mortality, and post-neonatal mortality were significantly higher in group 2 and group 3 than in group 1; the RRs of PTB, FGR, LBW, Apgar score <7, fetal mortality, neonatal mortality, and post-neonatal mortality ranged from 1.08 to 3.86 in group 2 and group 3 compared with group 1 (Table 3). The rates of hyaline membrane disease and assisted ventilation (< or > = 30 minutes) were also significantly higher in group 3 than in group 1 (Table 3). However there were no statistically significant differences in the rates of congenital anomalies among the three study groups (Table 3).

**Table 3:** Comparison of fetal and neonatal mortality and morbidity among three study groups in twins in United States

	Partner information available (N = 129651)	Partner information partly missing (N = 3018)		Partner information totally missing (N = 19564)	
	
	% (95%CI)	% (95%CI)	RR#	% (95%CI)	RR#
General condition					
PTB (<37W)	53.5 (53.2 – 53.8)	57.8 (56.0 – 59.7)*	1.08	59.6 (58.9 – 60.3)*	1.11
FGR (<10 percentiles z score)	8.26 (8.10 – 8.41)	10.9 (9.72 – 12.0)*	1.32	12.9 (12.4 – 13.4)*	1.56
LBW (<2500 g)	51.4 (51.1 – 51.7)	60.0 (58.2 – 61.7)*	1.17	64.6 (63.9 – 65.3)*	1.26
Apgar score (<7)	3.23 (3.13 – 3.32)	5.14 (4.35 – 5.92)*	1.59	6.45 (6.11 – 6.79)*	2.00
Fetal mortality	0.85 (0.81 – 0.89)	3.28 (2.65 – 3.90)*	3.86	1.59 (1.43 – 1.75)*	1.87
Neonatal mortality	1.92 (1.85 – 2.00)	3.83 (3.12 – 4.53)*	1.99	3.90 (3.62 – 4.17)*	2.03
Post-neonatal mortality	0.46 (0.43 – 0.50)	0.95 (0.59 – 1.32)*	2.07	1.12 (0.97 – 1.27)*	2.43
Abnormal conditions of the newborn					
Anemia (HGB.<13)	0.40 (0.36 – 0.43)	0.35 (0.13 – 0.57)	0.88	0.46 (0.36 – 0.55)	1.15
Birth injury	0.16 (0.14 – 0.18)	0.18 (0.02 – 0.33)	1.13	0.17 (0.11 – 0.23)	1.06
Hyaline membrane disease	3.28 (3.18 – 3.38)	2.88 (2.27 – 3.49)	0.88	3.87 (3.60 – 4.14)*	1.18
Meconium aspiration syndrome	0.09 (0.07 – 0.11)	0.11 (0.01 – 0.22)	1.22	0.09 (0.05 – 0.13)	1.00
Assisted ventilation (<30 minutes)	3.45 (3.35 – 3.55)	2.67 (2.08 – 3.26)*	0.77	3.87 (3.60 – 4.14)*	1.12
Assisted ventilation (> = 30 minutes)	3.82 (3.71 – 3.92)	3.23 (2.58 – 3.88)	0.85	5.04 (4.73 – 5.35)*	1.32
Seizures	0.07 (0.05 – 0.08)	0.18 (0.02 – 0.33)	2.57	0.10 (0.06 – 0.15)	1.43
Congenital anomalies					
Central nervous system anomalies	0.15 (0.13 – 0.18)	0.20 (0.04 – 0.36)	1.33	0.14 (0.09 – 0.19)	0.93
Circulatory / respiratory anomalies	0.43 (0.39 – 0.46)	0.60 (0.32 – 0.87)	1.40	0.45 (0.36 – 0.54)	1.05
Digestive system anomalies	0.11 (0.09 – 0.13)	0.17 (0.02 – 0.31)	1.55	0.11 (0.07 – 0.16)	1.00
Urogenital system anomalies	0.21 (0.19 – 0.24)	0.23 (0.06 – 0.40)	1.10	0.17 (0.11 – 0.23)	0.81
Musculoskeletal / integumental anomalies	0.38 (0.34 – 0.41)	0.43 (0.20 – 0.66)	1.13	0.40 (0.31 – 0.49)	1.05
Chromosomal anomalies	0.10 (0.09 – 0.12)	0.17 (0.02 – 0.31)	1.70	0.08 (0.04 – 0.12)	0.80

** P < 0.05 for group 2 or group 3 compared with group 1; #: relative risk compared with group 1.

## Discussion

Our large population based study found that the risks of fetal and infant mortality, low birth weight, preterm birth, fetal growth restriction, Apgar score <7, and the need for mechanical ventilation were increased in infants born to mothers with missing information on partner's age or race, especially when both variables of the partner were missing. The women reported no information (or reported only part of the information) on partners appear to have higher prevalence / incidence of most of the maternal morbidity and obstetric complications than in mothers with available information on partners. The poorer pregnancy outcomes observed in women with partner's information been partly and (especially) totally missing than in women with available information on partners were due largely to known important socioeconomic factors for adverse pregnancy outcomes. Many of these women were teenagers, black race, unmarried, with low education, high frequency of cigarette smoking, and inadequate prenatal care services. Teenagers [17], black race [18], unmarried [19], low education [3], cigarette smoking [4], and inadequate prenatal care services [20] are known risk factors of adverse pregnancy outcomes.

Our findings that the rates of FGR, LBW, Apgar score <7, hyaline membrane disease, and assisted ventilation were significantly higher in the group with paternal information totally missing than the group of only partly missing suggest that the totally missing group is a particularly high risk group, whereas in the partly missing group, the missing may have occurred in randomly.

Exceptions did occur, however. For example, the occurrences of several maternal medical conditions, such as cardiac disorders, diabetes, and RH sensitization, were actually lower in women with no available information on partners (group 2 and 3) than women with available information on partners (group 1). This may, in part, be due to the younger maternal age in groups 2 and 3 than in group 1. The risk of cardiac disorders and diabetes increases rapidly with advancing age [21,22]. Younger mothers are less likely to have had previous pregnancy and therefore less chance for RH sensitization [23]. The rates of obstetric interventions, including cesarean section, vacuum / forceps, and induction delivery, were lower in groups 2 and 3 than in group 1, which could be explained by the younger age of this group as cesarean section is less common in young women [24]. The rates of placenta previa and cephalopelvic disproportion were lower in women in groups 2 and 3 than in group 1, this could be explained by the younger age and lower parity of these groups as placenta previa and cephalopelvic disproportion are less common in younger and lower parity women [25,26]. We do not know why the rates of various congenital anomalies were not higher in infants born to mothers with no available information on fathers, despite their much higher risks of fetal and infant mortality and neonatal morbidity. Our study was based on birth certificates, and the diagnosis of many birth defects may be difficult at birth, especially when there is a lack of access to quality perinatal care services such as high resolution ultrasound [27], although differences in maternal age may play a role here.

Our study examined the difference of adverse pregnancy outcomes among three study groups in twins only. It is biologically plausible to apply the study findings to singletons and higher order of multiple pregnancies as well, although the magnitude of the differences may be smaller in singletons and larger in higher order of multiple pregnancies than in twins.

Since in the database, there was no indication why a particular variable was not recorded, we can only speculate the reason of missing partner's information. Corresponding variables (i.e., age and race) for the mothers, including those mothers with missing information on partners, were completely recorded. These women may have no relationship or only remote relationship with their partners, as suggested by the extraordinarily high unmarried rates (61% for women missing one variable on their partners and 96% for women missing both variables on their partners). As a result, they may have difficulty or for unknown reasons they may be reluctant to provides partner's information. The paternal information missing may be a comprehensive measure and may be related with age, education, socioeconomic status, race, religion background, or even the reporting quality in different place. Our study only described the association between the paternal information missing and the adverse pregnancy outcomes. Exploration of the reasons of missing partner information and its relationship with adverse pregnancy outcomes require in-depth analysis of data with relevant information.

## Conclusions

Women with no available information (or partly) on partners appear to have higher risks of developing adverse pregnancy outcomes. For this reason, extra attention for these women and their offspring by health care providers and public health workers is needed.

## Competing interests

The authors declare that they have no competing interests.

## Authors' contributions

Hongzhuan Tan carried out the data analysis and drafted the manuscript. Shi Wu Wen designed the study and helped in results interpretation. Mark Walker participated in the sequence alignment and participated in the results interpretation. Kitaw Demissie participated in the study design and results interpretation.

## Pre-publication history

The pre-publication history for this paper can be accessed here:


